# Aging, Immunity, and COVID-19: How Age Influences the Host Immune Response to Coronavirus Infections?

**DOI:** 10.3389/fphys.2020.571416

**Published:** 2021-01-12

**Authors:** Varnica Bajaj, Nirupa Gadi, Allison P. Spihlman, Samantha C. Wu, Christopher H. Choi, Vaishali R. Moulton

**Affiliations:** ^1^Division of Rheumatology and Clinical Immunology, Department of Medicine, Beth Israel Deaconess Medical Center, Harvard Medical School, Boston, MA, United States; ^2^School of Medicine, Boston University, Boston, MA, United States

**Keywords:** aging, immunity, coronavirus, SARS-CoV, COVID-19, infection, immune response

## Abstract

The novel coronavirus severe acute respiratory syndrome coronavirus 2 causing the Coronavirus disease (COVID-19) pandemic has ravaged the world with over 72 million total cases and over 1.6 million deaths worldwide as of early December 2020. An overwhelming preponderance of cases and deaths is observed within the elderly population, and especially in those with pre-existing conditions and comorbidities. Aging causes numerous biological changes in the immune system, which are linked to age-related illnesses and susceptibility to infectious diseases. Age-related changes influence the host immune response and therefore not only weaken the ability to fight respiratory infections but also to mount effective responses to vaccines. Immunosenescence and inflamm-aging are considered key features of the aging immune system wherein accumulation of senescent immune cells contribute to its decline and simultaneously increased inflammatory phenotypes cause immune dysfunction. Age-related quantitative and qualitative changes in the immune system affect cells and soluble mediators of both the innate and adaptive immune responses within lymphoid and non-lymphoid peripheral tissues. These changes determine not only the susceptibility to infections, but also disease progression and clinical outcomes thereafter. Furthermore, the response to therapeutics and the immune response to vaccines are influenced by age-related changes within the immune system. Therefore, better understanding of the pathophysiology of aging and the immune response will not only help understand age-related diseases but also guide targeted management strategies for deadly infectious diseases like COVID-19.

## Introduction

From infecting exotic wild animals to royalty, the novel coronavirus severe acute respiratory syndrome coronavirus 2 (SARS-CoV-2) has ravaged the world with the coronavirus disease 2019 (COVID-19) pandemic (Guan et al., 2020; Shi et al., 2020). While SARS-CoV-2 is related and similar in structure to its older relatives SARS-CoV and Middle eastern respiratory syndrome coronavirus (MERS-CoV) of the Coronavirus family, which caused the SARS and MERS epidemics, respectively, this novel strain has rapidly spread across continents causing the worst pandemic of the 21st century. A striking feature of COVID-19 is the demographics of the populations afflicted. While men suffer worse outcomes and higher mortality rates than women, and individuals of black and hispanic race/ethnic minorities are disproportionately affected (Gadi et al., 2020; Kopel et al., 2020; Muñoz-Price et al., 2020), most notably, elderly individuals >60 years of age have been most severely afflicted as evidenced by the significantly high morbidity and mortality in this group (Santesmasses et al., 2020). A majority of cases and deaths in every country worldwide involves the aging population, and numbers correlate with increasing age. In addition, the presence of comorbidities including obesity, diabetes, hypertension, cardiovascular, and lung disease and cancer accompanied by immunocompromised states and tissue damage predispose to the significantly higher rates of mortality in this group.

Severe acute respiratory syndrome coronavirus 2 is a large (27.9–31 kb) enveloped positive sense single stranded RNA virus, which enters the human host most commonly through the respiratory tract and attaches to cells via its spike (S) protein using the angiotensin converting enzyme 2 (ACE2) receptor on alveolar epithelial type II pneumocytes in the lung (Zhang H. et al., 2020). Once inside cells, it replicates and new viral particles release from cells and invade neighboring cells and extracellular spaces and cause tissue damage leading to clinical signs and symptoms. Besides the lung, the ACE2 receptor is highly expressed in epithelial cells of the gastrointestinal tract and in other organs including the kidneys, vascular endothelial cells, and other tissues including high expression in adipose tissues (Hamming et al., 2004; Al Heialy et al., 2020; Al-Benna, 2020; Albini et al., 2020; Gu et al., 2020; Li et al., 2020; Thum, 2020; Verdecchia et al., 2020; Verma et al., 2020). Accordingly, the clinical presentation can vary with respiratory symptoms being most common such as cough, shortness of breath, and fever, but GI symptoms such as diarrhea, nausea vomiting are not uncommon. Recently clinical signs of vascular endothelial dysfunction and abnormal coagulopathy-related manifestations have been reported leading to thromboembolism and strokes (Oxley et al., 2020). Severe disease complications include pneumonia and acute respiratory distress syndrome (ARDS) requiring mechanical ventilation and can be fatal.

The normal immune response to viral infections including coronaviruses involves both the innate and adaptive immune systems. Innate mechanisms include the recognition of viral nucleic acids via the toll-like receptors (TLR) expressed by dendritic cells (DC) and macrophages, followed by intracellular signaling and production of antiviral type I interferon (IFN) in antiviral defense. Natural killer (NK) cells are important in the early antiviral defense mechanisms. In addition, monocyte-macrophage mediated production of proinflammatory cytokines IL-6, IL-1β, tumor necrosis factor (TNF) and chemokines enable the recruitment of neutrophils and other inflammatory immune cells to the site of infection. The adaptive immune response is initiated upon viral antigen presentation to CD4 helper T cells and CD8 cytotoxic T cells. Cytokines such as IFN-γ are important to activate macrophages and cellular immunity, while antigen-specific CD8 T cells are important for killing viral infected cells. CD4 helper T (Th) cells are important in providing cognate help to B cells, which undergo class switching, and somatic hypermutation to shift production of antibodies from the IgM to more specific high affinity neutralizing IgG isotypes. Notably, the immune response is a double edged sword which on one hand mediates protective immunity and is necessary, but on the other hand, excess and inappropriate production of inflammatory cytokines can lead to cytokine storm syndrome (Zhou Y. et al., 2020) causing organ immunopathology leading to complications and death, and this scenario could not be more true for COVID-19.

**FIGURE 1:**
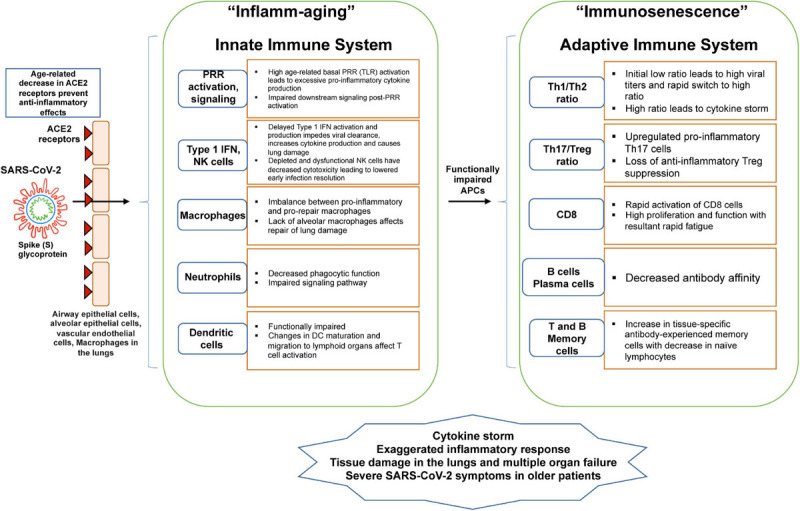
Schematic shows age-related changes in the innate and adaptive immune system with relevance to COVID-19. As the SARS-CoV-2 enters parenchymal cells expressing the ACE2 receptor, it activates PRR of the innate immune system. With aging, there is an imbalance in cellular functions and signaling, decreasing innate activation of an already weakened adaptive immune system. These imbalances lead to “inflammaging” from proinflammatory innate immune responses and immunosenescence of the adaptive immune response.

As with every system in the body, natural aging is accompanied by progressive biological changes in the immune system (Figure 1), some of which lead to its declining functions as evidenced by increased susceptibility to respiratory infections such as influenza and novel coronaviruses. On the other hand, age-related immune-mediated inflammation or inflamm-aging, and associated inflammatory diseases increase with aging (Franceschi et al., 2000; Shaw et al., 2013; Weyand and Goronzy, 2016; Fuentes et al., 2017; Fulop et al., 2018). These changes in concert with comorbidities together render older individuals vulnerable to latent or novel infections and lead to the observed increases in morbidity and mortality of COVID-19. In younger individuals, a larger repertoire of naive immune cells endow the ability to fight new infections and respond to foreign antigens successfully thus resulting in milder forms of the disease or even asymptomatic infection, as is observed in a majority of younger individuals who test positive for the novel coronavirus. Increased combined diversity of the antigen receptors in T and B lymphocytes enables a larger robust pool of precursors able to recognize a wider range of foreign organisms and better able to fend off new diseases such as those caused by the novel coronavirus.

**FIGURE 2:**
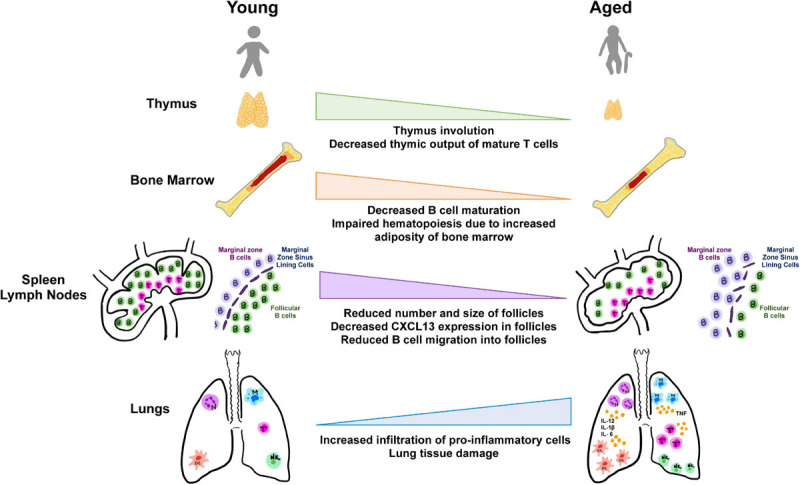
Aging and tissues in immunity. Aging is accompanied by the decline in function of lymphoid and non-lymphoid tissues involved in the host immune response. Degeneration of primary lymphoid organs lowers production of naive T and B lymphocytes which results in reduced migration to secondary lymphoid organs and to sites of antigen encounter. Additionally, the lungs and extrapulmonary organs are susceptible to accumulation of proinflammatory cells and mediators.

This review will focus on concepts of immunosenescence and inflamm-aging as they pertain to age-related physiologic changes in the immune system, and how these influence the host immune response to viral infections including coronaviruses. We discuss how aging impacts the innate and adaptive immune systems specifically focusing on cellular components and soluble secretory mediators such as antibodies and cytokines. We further discuss tissue-specific aspects of immunity related to aging including physiologic changes in lymphoid tissues and peripheral non-lymphoid tissue sites of infection such as the lung (Figure 2). Finally we review therapeutic and preventive vaccine approaches underway including antivirals and immune-modulating agents, and immune response to vaccines influenced by aging with relevance to COVID-19.

## Immunosenescence and Inflamm-Aging

Immunosenescence describes the age-associated shift in both innate and adaptive immune systems that leads to the reduced ability to fight novel infections and contributes to the development of a chronic state of inflammation (Stahl and Brown, 2015). These alterations of the immune system lead to higher rates of infection and disease. Changes observed in immunosenescence are not simply a decline of immune cells but reflect a more complex change in action across various immune responses. Several hallmarks are commonly characteristic of immunosenescence. The immune system is compromised in its ability to respond effectively to new antigens, linked to a decrease in peripheral naive T and B cells (Stahl and Brown, 2015). Accumulation of memory T cells is also associated with immunosenescence. The antigen load experienced within a lifetime coupled with decreased lymphopoiesis, decreases naive T cell populations and leads to an accumulation of memory cell subsets. Accumulation of CD4 and CD8 T cells that have lost the expression of costimulatory surface protein CD28 is associated with aging and this loss of CD28 is considered a key feature of senescent T cells (Weyand and Goronzy, 2016). These CD28null T cells express other markers including CD27, another co-stimulatory molecule, and are characterized by short telomeres, a lack of telomerase, and inhibitory signaling receptors. This shorter leukocyte telomere length is associated with stress and several stress and aging-related disorders (de Punder et al., 2019). CD28 signaling is necessary for optimal telomerase up-regulation, and experiments *in vitro* have demonstrated that loss of telomerase activity occurs concurrently with loss of CD28 expression in T cells (de Punder et al., 2019). Senescent cells often undergo changes in gene expression, making them less likely to undergo apoptosis, and many secrete molecular factors such as cytokines, which impact their surrounding microenvironment. Immunosenescence represents a remodeling of the immune system and reflects the plasticity of this system for change with age (Alves and Bueno, 2019).

Chronic low-grade inflammation, termed “inflamm-aging” is associated with immunosenescence, driven by a reduced ability to endure inflammatory triggers as well as an increased production of pro-inflammatory cytokines, acute phase proteins, and oxidative stressors (Aiello et al., 2019). The age-associated increase in the number of senescent cells in conjunction with the decrease in the immune system’s ability to remove these cells results in an environment rich with pro-inflammatory cytokines and reactive oxygen species (ROS) and further drives neighboring cells into senescence (Stahl and Brown, 2015). Inflammaging is associated with chronic stimulation of the innate immune system and increased secretion of inflammatory mediators as evidenced by increased proinflammatory NF-κB signaling (Salminen et al., 2018). However, under stimulation with *in vivo*/*vitro* exogenous antigens or mitogens, inflammaging is also associated with down-regulated innate immune responses, driven by regulatory subtypes of T and B cells as well as regulatory subtypes of macrophages, dendritic, NK, and type II NK T cells (Salminen, 2020). This chronic inflammatory environment therefore induces immunosuppression through downregulation of both innate and adaptive immune responses (Salminen et al., 2019). Myeloid-derived suppressor cells (MDSC) also regulate the differentiation and function of several other immune cell types to prevent inflammation (Salminen et al., 2019). MDSCs induce the differentiation of regulatory T cells and regulatory B cells, which both have immunosuppressive effects via secretion of anti-inflammatory cytokines such as IL-10 and TGF-β. It has been observed that the numbers of both MDSCs and regulatory T cells are significantly increased during the inflammaging process (Salminen et al., 2018). While the functions of these cells have not been fully elucidated, they have been associated with many aging-related morbidities such as infection, cancer, and autoimmune disease (Alves and Bueno, 2019). These cells may potentially have a role in the generation of immunosenescence. Additionally, it has been found that there are elevated plasma levels of interleukin (IL)-6, IL-1, and TNF in the elderly population (Martín et al., 2017). These factors lead to constant activation of immune response, leading to a constant, continued inflammatory response.

Several current theories exist to explain how immunosenescence occurs. The autoimmune theory describes immunosenescence as the result of production of autoantibodies secondary to age-associated thymus involution, which leads to a deficiency of naive T cells in addition to, increased activation of T cells to “neoantigens” (Fuentes et al., 2017). The immunodeficiency theory simply postulates immunosenescence as the impaired ability to mount defenses against new infections due to decreased availability of naive T cells (Fuentes et al., 2017). In addition, with age, there are decreased levels of plasma B cells but increased levels of circulating immunoglobulin from antibody-producing B lymphocyte cells with low antigen specificity. More specifically, this impaired ability to produce high affinity immunoglobulin molecules is the result of a shift from immunoglobulin production by naive B cells (IgD, IgM) to those produced by memory B cells (IgG, IgA) (Aiello et al., 2019). The deregulation theory proposes that immunosenescence results from changes to ratios of different expressed gene isoforms caused by changes in RNA processing with age resulting in atypical or abnormal secondary immune cell activation (Fuentes et al., 2017). Infection with latent viruses such as cytomegalovirus (CMV) has also been shown to potentially play a role in immunosenescence by amplification of aging-associated inflammation, and both CMV seropositivity and elevated CMV IgG antibody titers are associated with phenotypic/functional alterations to adaptive immunity and an increased risk for all-cause mortality (Solana et al., 2012). Studies to understand the full extent of its involvement are still underway.

While the numerous immunological correlates and consequences of COVID-19 have yet to be determined, it is clear that immune processes are involved. When in the lungs, SARS-CoV-2 induces cytokine storm and a strong immune response characterized by the increased presence of many inflammatory cytokines (Nieman, 2020). SARS-CoV-2 shares almost 80% RNA sequence homology with SARS-CoV, making it possible that it uses similar strategies to evade immune system responses as SARS-CoV (Felsenstein et al., 2020). The changes associated with immunosenescence leaves older adults particularly vulnerable to SARS-CoV-2 and more severe COVID-19 infection (Nikolich-Zugich et al., 2020). T lymphocytes, particularly CD4+ T cells, CD8+ T cells, and B cell activation are critical in the host defense against viral infections (Felsenstein et al., 2020). Thus, the depletion of these cells combined with the expression of pro-inflammatory cytokines likely play a role in triggering the hyper-inflammatory reaction seen among COVID-19 patients.

## Innate Immunity

### SARS-CoV-2 Entry and ACE2 Receptors

Coronaviruses use the surface spike (S) glycoprotein to enter human host cells via the ACE2 receptor on the host cells (Nikolich-Zugich et al., 2020; Wan et al., 2020). In humans, three common strains of coronaviruses, SARS-CoV, Human Coronavirus NL-63 (NL63), and SARS-CoV-2 target airway epithelial cells, alveolar epithelial cells, vascular endothelial cells, and macrophages in the lungs as they express abundant ACE2 receptors (Tay et al., 2020; Verdecchia et al., 2020). As compared to SARS-CoV, the SARS-CoV-2 evolved by mutating part of its S glycoprotein to have a 10–20× higher affinity for the ACE2 receptor (Nikolich-Zugich et al., 2020; Wrapp et al., 2020). The main respiratory cells targeted by SARS-CoV and SARS-CoV-2 for ACE2 receptor-mediated entry are type 2 pneumocytes and ciliated bronchial epithelial cells (Albini et al., 2020). ACE2 receptors are also present in the heart (cardiomyocytes and pericytes), gut (enterocytes of the small intestine and colon), kidneys (renal tubular and intestinal epithelial cells), testis (spermatogonia, Leydig cells, and Sertoli cells), brain (neurons and glial cells), arterial smooth muscle cells, arterial and venous endothelial cells, myofibroblasts and adipocytes, that can be affected in patients with co-morbidities such as cardiovascular disease, chronic obstructive pulmonary disease, pulmonary hypertension, and diabetes (Hamming et al., 2004; Xia and Lazartigues, 2008; Albini et al., 2020; Gu et al., 2020; Li et al., 2020; Thum, 2020; Verdecchia et al., 2020; Verma et al., 2020). By binding to the ACE2 receptor on endothelial cells, SARS-CoV-2 can lead to endothelial cell inflammation, vasculitis, disseminated intravascular coagulation, and thromboembolism (Albini et al., 2020). SARS-CoV-2 can also infect enterocytes via ACE2 causing diarrhea and associated pain and cramping (Gu et al., 2020).

Higher ACE2 receptor expression levels were found in the nasal epithelium of young adults less than 60 years compared to those in children (Bunyavanich et al., 2020). This higher ACE2 expression in young adults may predispose them to an increased risk for contracting coronavirus infections. Accordingly, based on epidemic trajectories mapped from epidemiological data from South Korea, the highest numbers of daily new SARS-CoV-2 positive cases were among younger individuals in the 20–39 and 40–59 year age groups compared to people over 60 years (Yu et al., 2020). However, while it has been coopted as the entry point for the SARS-CoV-2 virus on host cells, the ACE2 enzyme also plays a critical anti-inflammatory role in the renin-angiotensin-aldosterone-system (RAAS) signaling pathway by converting the proinflammatory angiotensin 2 to anti-inflammatory angiotensin 1–7 (Rodrigues Prestes et al., 2017; Albini et al., 2020; AlGhatrif et al., 2020). Moreover, aging has been associated with decline in levels of ACE2 expression in murine models and humans (Chen J. et al., 2020; Xie et al., 2006; Yoon et al., 2016). Specifically, aging-related decrease in ACE2 expression levels were observed in lung epithelial cells of aged rats compared to young rats (Xie et al., 2006), and in the thoracic aorta of old versus young mice (Yoon et al., 2016). Furthermore, in a bioinformatic analysis of publicly available human genomics and transcriptomics gene expression data, age-associated decrease in ACE2 expression levels was observed in multiple tissues including blood, kidneys, and adrenal glands, and also found to be lower in type 2 diabetes patients compared to healthy individuals (Chen J. et al., 2020). Therefore, a decrease in ACE2 receptor expression coupled with aging-related immune inflammation and comorbidities may compromise the anti-inflammatory response and predispose older individuals to exaggerated inflammatory responses, which is one of the hallmarks of COVID-19 (Ciaglia et al., 2020; Verdecchia et al., 2020). Binding of SARS-CoV-2 to ACE2 receptors further lowers the ACE2 expression (Banu et al., 2020; Verdecchia et al., 2020), which increases the angiotensin 2 activity that contributes to an acute exaggerated pro-inflammatory response (Kuba et al., 2005; AlGhatrif et al., 2020; Ciaglia et al., 2020; Verdecchia et al., 2020). However, there has been some controversy regarding the variability in ACE2 expression with aging as ACE2 expression in the lungs were shown to decrease in aged rats (Xie et al., 2006) but the expression levels in aged male and female mice (Nozato et al., 2019) and humans have been variable based on the cohort (Fernández-Atucha et al., 2017; Li et al., 2020). Altogether, despite the lower rates of infection in older individuals, the prognosis of COVID-19 may be worse due to a decrease in ACE2 expression levels and a decrease in anti-inflammatory response.

A decrease in ACE2 expression has been reported in individuals with hypertension and diabetes (Li et al., 2020; Verdecchia et al., 2020) making them susceptible to increased inflammation via the angiotensin 2 pro-inflammatory pathway, while treatment with ACE inhibitors (ACEI) and angiotensin receptor blockers (ARB) tends to increase ACE2 expression (Li et al., 2017). This ACE2 upregulation can be physiologically restorative given its anti-inflammatory roles, however, may predispose them to a greater risk of SARS-CoV-2 infection (Fang et al., 2020; Li et al., 2020). Because ACE2 is upregulated in patients with diabetes and cardiovascular disease who are treated with ACEI and ARBs, this creates a controversy regarding these treatments (Albini et al., 2020; AlGhatrif et al., 2020; Fang et al., 2020; Onweni et al., 2020). Hence, the treatment of older hypertensive and diabetic individuals with ACEIs and ARBs has been a dilemma (AlGhatrif et al., 2020). However, the WHO and others suggest continuing the use of these drugs in COVID-19 patients since no risk has been shown,^1^ including in the recent first randomized BRACE Corona trial,^2^ and also because of their beneficial role (Meng et al., 2020; Yan et al., 2020).

In addition to ACE2 receptors, dipeptidyl peptidase 4 (DPP4) might serve as a binding target for SARS-CoV-2 entry (Solerte et al., 2020; Valencia et al., 2020). DPP4 expression is enhanced in type 2 diabetic patients, obese individuals and senescent cells which increases proliferation of human smooth muscle cells and upregulation of pro-inflammatory cytokines such as MCP-1, IL-6, and IL-8 via NF-kB activation (Röhrborn et al., 2015; Valencia et al., 2020), thus potentially leading to worse outcomes of SARS-CoV-2 in diabetic patients. Therefore, the use of DPP4 inhibitors may be beneficial as they significantly lower the levels of IL-6, which plays a major role in the inflammatory response in SARS-CoV-2 patients (Solerte et al., 2020; Valencia et al., 2020).

### Innate Pro-inflammatory Responses

Once the SARS-CoV-2 enters the host cells via the ACE2 receptors, it actively replicates followed by release of viral particles (Tay et al., 2020). This causes the host cell to undergo pyroptosis and release damage-associated molecular patterns (DAMPs) such as ATP, nucleic acid, ASC oligomers, and cytokines (Lee et al., 2020; Tay et al., 2020). These DAMPs trigger the neighboring epithelial cells, endothelial cells, and alveolar macrophages to generate pro-inflammatory cytokines and chemokines such as IL-6, IP-10, macrophage inflammatory protein (MIP) 1-alpha and 1-beta (Lee et al., 2020; Tay et al., 2020). These proteins further attract monocytes, macrophages, and T cells, which also produce IFN-γ, creating a pro-inflammatory feedback loop (Tay et al., 2020). However, in case of a defective immune response, there is an increased build up of immune cells in the lungs, overproduction of proinflammatory cytokines leading to tissue damage in the lungs, ARDS and multi-organ damage due to the resulting cytokine storm which can be fatal (Tay et al., 2020). Excessive inflammation and cytokine storm are characteristics of cytokine release syndrome, which is elicited by SARS-CoV-2 in patients with dysregulated immune responses (Acharya et al., 2020; Tay et al., 2020). The innate immune system tends to dysregulate with aging and acquires proinflammatory phenotypes accompanied by tissue inflammation (Acharya et al., 2020).

There are several patterns of immune responses to coronaviruses, primarily SARS-CoV and SARS-CoV-2 (Du and Yuan, 2020; Nieman, 2020; Nikolich-Zugich et al., 2020). Severe symptoms are associated with intense pro-inflammatory responses and cytokine storm that can cause tissue damage especially in the lungs (Nieman, 2020; Nikolich-Zugich et al., 2020). In SARS-CoV, the non-survivors displayed a state termed as “stuck in innate immunity” in which there was an increased innate activation of IFN-α and IFN-γ, CXCL10, CCL2, and downstream IFN stimulated genes, but a lack of antibody production (Nikolich-Zugich et al., 2020). This state was more prevalent in the older individuals (Nikolich-Zugich et al., 2020). Some studies have shown that SARS-CoV-2 may induce an early activation of the adaptive immune response system, which may interfere with the innate immune response to eliminate the virus quickly (Du and Yuan, 2020). This may lead to an overreaction of the immune system causing a cytokine storm that could be damaging to the tissues and possibly lead to two waves of the disease (Du and Yuan, 2020).

With age, the innate inflammatory responses gain in intensity and duration leading to many inflammatory diseases such as rheumatoid arthritis, cardiovascular disease, and neurodegenerative disease and increased tissue damage (Weyand and Goronzy, 2016). This phenomenon is known as “inflamm-aging,” which is coupled with other changes in the immune system with age known as “immunosenescence” (Fulop et al., 2018). This age-associated chronic basal inflammation is in part due to the continuous engagement of pattern recognition receptor (PRRs) on innate immune cells with pathogen associated molecular patterns (PAMPs) via reactivation of latent viral infections or the release of endogenous DAMPs that are released from senescent and necrotic cells (Shaw et al., 2013; Frasca et al., 2017; Rea et al., 2018). TLRs are the main PRRs that are highly expressed on monocytes, macrophages, neutrophils, DC, and some lymphocytes (Rea et al., 2018; Moreno-Eutimio et al., 2020; Onofrio et al., 2020; Plenge, 2020; Sallenave and Guillot, 2020). TLRs that play a major role in SARS-CoV-2 are TLR-7 and TLR-8, which sense the ssRNA of SARS-CoV-2 and are expressed in the endosomal compartments of respiratory epithelial cells (Moreno-Eutimio et al., 2020; Onofrio et al., 2020; Plenge, 2020; Sallenave and Guillot, 2020). TLR-7 and TLR-8 induce the production of IFN-regulated cytokines and production of pro-inflammatory cytokines, respectively (Moreno-Eutimio et al., 2020; Onofrio et al., 2020). The stimulator of IFN gene (STING) pathway, involved in the production of IFNs and NF-kB in response to cytosolic DNA and RNA viruses, is also believed to be involved in SARS-CoV-2 infection and is upregulated in senescent cells (Berthelot and Lioté, 2020). Innate immune cell migration and signaling events downstream of PRR activation are impaired with aging leading to increased cytokine secretion and dysregulation, also known as a cytokine storm (Shaw et al., 2013; Onofrio et al., 2020). Age-related alterations in TLR protein expression in innate immune cells can induce cytokine production (Shaw et al., 2013; Onofrio et al., 2020). For example, a lowered surface expression of TLR1 associated with lowered TLR1/TLR2 can induce cytokine production in human monocytes (Shaw et al., 2013; Onofrio et al., 2020). This defect is primarily post-translational that worsens with age (Shaw et al., 2013; Onofrio et al., 2020). Over-production of cytokine reflects high basal TLR activation, which cannot be further activated in response to a pathogen, contributing to a failure of innate immune system response (Shaw et al., 2013; Onofrio et al., 2020).

Age-related impairment in PRR signaling can affect macro-autophagy and vice versa (Shaw et al., 2013; Rea et al., 2018). Defects in either can stimulate the inflammatory cascade (Shaw et al., 2013; Rea et al., 2018). Autophagy is a process that removes damaged proteins and large aggregates to regenerate new and healthy cells. These autophagic responses to intracellular pathogens are impaired in older individuals (Shaw et al., 2013; Rea et al., 2018). Impaired autophagy can in turn affect PRR signaling via increased ROS, which increases with age (Shaw et al., 2013; Rea et al., 2018). ROS is unregulated and overproduced as a by-product of mitochondrial energy production, active immunological phagocytic processes and prostaglandin pathways through COX enzyme production that begin to dysfunction with age (Rea et al., 2018). In addition to changes in redox balance, age-related increase in senescent cells, senescence-associated secretory phenotype (SASP), excess oxidative state, DNA damage and decline in successful autophagy can trigger inflammasome by stimulating an inflammatory cascade involving NF-κB, IL-1α, TGF-β, and IL-6 pathways (Shaw et al., 2013; Rea et al., 2018). Excessive secretion of ROS, along with the secretion of other proteases, can cause an uninhibited infiltration of inflammatory cells damaging the lungs in addition to the damage caused by SARS-CoV-2 virus (Rea et al., 2018; Tay et al., 2020).

The anti-viral innate immune response also includes soluble components of the complement and coagulation system, such as mannose-binding lectin (MBL), and natural antibodies such as IgM, and IgA (Matricardi et al., 2020). Reduction of these mediators may contribute to the increased susceptibility of older people to SARS-CoV-2. Age-dependent decrease of natural IgM (Muthana and Gildersleeve, 2016) and MBL (Terai et al., 1993; Tomaiuolo et al., 2012) have been described. MBL is higher in those under 20 years compared to those over 20, and is noted to decrease with age (Tomaiuolo et al., 2012), and an age-related decline in IgM begins between the 40s and 50s (Terai et al., 1993; Matricardi et al., 2020). Therefore, the decrease in MBL and IgM with age may compromise the anti-viral innate immune response and increase the susceptibility of older individuals to COVID-19 (Terai et al., 1993; Tomaiuolo et al., 2012; Muthana and Gildersleeve, 2016; Matricardi et al., 2020).

### Type I IFN and NK Cells

During a pathogenic invasion, PAMPs and DAMPs activate PRRs that initiate a signaling cascade, which leads to the expression of type I IFN and other inflammatory factors (Acharya et al., 2020; Nikolich-Zugich et al., 2020). An early activation and production of type I IFN is protective in antiviral defense (Acharya et al., 2020; Nikolich-Zugich et al., 2020). Type I IFN acts as an immediate antiviral response via autocrine and paracrine type I IFN receptor (IFNAR) signaling to restrict viral replication and spread (Acharya et al., 2020; Nikolich-Zugich et al., 2020). However, a delay in production can lead to tissue damage and inflammatory cytokine storm (Acharya et al., 2020; Nikolich-Zugich et al., 2020). This delay in type I IFN production increases with age and was seen in older individuals with SARS-CoV (Acharya et al., 2020; Nikolich-Zugich et al., 2020). Additionally, the evolved immune evasive mechanisms of the SARS-CoV virus prevent early induction of the type I IFN, which was initially assumed to be the case in SARS-CoV-2 (Acharya et al., 2020; Nikolich-Zugich et al., 2020).

In addition to the delay in local IFN responses, which impedes viral clearance and leads to the development of cytokine release syndrome, type I IFN are minimally detected in patients with severe SARS-CoV-2 symptoms (Aiello et al., 2019; Acharya et al., 2020). Due to this impaired IFN production, there is an imbalance between proinflammatory macrophages and reparative airway macrophages (Aiello et al., 2019; Acharya et al., 2020). Additionally, other immune cells such as NK cells are regulated by IFN during coronavirus infections, which are also depleted and dysfunctional in patients with severe SARS-CoV-2 symptoms (Aiello et al., 2019; Acharya et al., 2020). Impaired NK cell function is associated with higher rates of infection and mortality in older adults (Shaw et al., 2013). As per *in vivo* models, cytotoxicity of NK cells induced by type I IFN decreases in aged mice (Plett and Murasko, 2000; Shaw et al., 2013). Additionally, IFN production from plasmacytoid DCs declines with age in response to virus (Agrawal, 2013). Therefore, the impaired and delayed production of type I IFN in older individuals along with impaired production of NK cells affect the first-line antiviral response to SARS-CoV-2 invasion and lowers the chances of infection resolution at an earlier stage as compared to younger individuals (Shaw et al., 2013; Aiello et al., 2019; Acharya et al., 2020).

### Neutrophils, Macrophages, and Dendritic Cells

Neutrophils and macrophages are phagocytic cells of the first-line of defense whose phagocytic functions decrease with aging (Shaw et al., 2013; Jackaman et al., 2017). Furthermore, age-related changes in macrophages and neutrophils contribute to chronic low-grade inflammation and dysregulation of immunosuppression mediated by macrophages (Shaw et al., 2013; Jackaman et al., 2017). In older individuals, neutrophils migrate inaccurately and spread further in response to stimuli due to constitutive activation of the PI3K pathway, have reduced phagocytosis and decreased intracellular killing activity (Shaw et al., 2013; Jackaman et al., 2017). Age-related defects in neutrophils are primarily due to impaired signal transduction pathways such as impaired anti-apoptotic responses to GM-CSF mediated through the JAK-STAT tyrosine kinase and PI3K-AKT pathways (Shaw et al., 2013; Jackaman et al., 2017). Even though many viruses can stimulate the production of neutrophil extracellular traps (NETs) from neutrophils, there is no significant evidence on the role of neutrophils or neutrophil NETs in SARS-CoV-2 pathogenesis (Mozzini and Girelli, 2020).

In healthy elderly adipose and hepatic tissue, the macrophages are highly present as a pro-inflammatory M1 phenotype and as an immunosuppressive M2 phenotype in elderly lymphoid tissues, lung, and muscle (Jackaman et al., 2017). Macrophages can be easily activated in older individuals as a result of accumulation of immune complexes, elevated cytokines, hormones, free fatty acid, oxidized low-density lipoproteins and immunoglobulins (Acharya et al., 2020). This activation promotes low-grade inflammation negatively affecting the effectiveness of immune response (Acharya et al., 2020). In patients with SARS-CoV-2, there is an imbalance between proinflammatory macrophages and pro-repair airway macrophages due to impaired IFN production (Acharya et al., 2020). As a result of age-related decrease in IL-12 production by DCs and microenvironment alterations from changes in splenic marginal zones, antigen presenting cells become functionally impaired (Shaw et al., 2013). Alterations in the lung microenvironment also lead to changes in DC maturation and migration to lymphoid organs, thus affecting T cell activation in SARS-CoV-2 patients (Tay et al., 2020).

### Comorbidities and Innate Immune Responses

Elderly patients (over the age of 60) and patients with comorbidities experience more severe symptoms of SARS-CoV-2 partially due to hyperactive chronic innate inflammatory responses as well as structural and functional changes in organs (Frasca et al., 2017). Obesity increases the complications associated with the virus and aging in part due to structural/functional impairments (Iacobellis et al., 2020a; Malavazos et al., 2020) and also because visceral fat may serve as a reservoir for the virus and amplify inflammatory responses (Ryan and Caplice, 2020). Obese tissues can affect the mechanics of the respiratory system by reducing the compliance and lung volumes (Littleton, 2012) and pose as a risk factor for asthma (Tashiro and Shore, 2019). Computed tomographic (CT) imaging showed evidence of increased fatty liver and epicardial adipose tissues in older COVID-19 patients who were critically ill (Iacobellis et al., 2020b), and also in younger patients under 40 with severe disease compared to those with mild disease (Deng et al., 2020), suggesting obesity as a potential predictor of COVID-19 severity. Adipose tissues also serve as an endocrine organs and secrete hormones like leptin, adiponectin, and inflammatory cytokines such as IL-6 and TNF (Banerjee et al., 2020). ACE2 expression levels are higher in visceral adipose tissue compared to subcutaneous fat (Zhang et al., 2018), and since obese and diabetic patients have more adipose tissue, they have increased numbers of ACE2 expressing adipocytes, potentially increasing their susceptibility to an elevated viral load in the adipose tissue (Banerjee et al., 2020), which could lead to increased severity of SARS-CoV-2 infection (Fajnzylber et al., 2020). Aging is also associated with metabolic dysfunction in correlation with dysfunction of adipose tissue, as evidenced by correlation between mitochondrial and arterial dysfunction in epididymal white adipose tissue and metabolic dysfunction in aged B6D2F1 mice (Donato et al., 2014). Additionally in obese individuals, innate immune cells such as macrophages, NK cells, and neutrophils as well as adaptive immune cells infiltrate the adipose tissue and induce inflammatory responses via pro-inflammatory cytokine secretion, metabolic switches, and phenotypic and functional changes (Frasca et al., 2017). Chronic low-grade inflammation promoted by excess nutrients and elevated pro-inflammatory cytokines can cause insulin resistance in both mice and humans, which can in turn produce systemic and local inflammation (Frasca et al., 2017). Furthermore, obesity is a risk factor for endothelial dysfunction (Engin, 2017) and when combined with SARS-CoV-2 induced endothelial cell injury, could potentially exacerbate endothelial dysfunction leading to further complications (Amraei and Rahimi, 2020).

The combined age-related dysfunctional patterns of different aspects of the innate immune inflammatory responses prevent resolution of this viral infection (Frasca et al., 2017). Hence, older individuals over 60 years and individuals with co-morbidities are at a higher risk for SARS-CoV-2 severity as they are more likely to develop a defective immune response and suffer higher rates of morbidity and mortality.

## Adaptive Immunity

### CD4 T Cells

CD4 T helper cells are a key lymphocyte population of the adaptive immune system. By recognizing MHC Class II molecules on antigen-presenting cells, they are the first responders of adaptive immunity and their function through life is critical for prevention of host morbidity and mortality. They are especially important in discussions of COVID-19, as their effector roles for viral protection must be balanced with their regulatory roles to prevent host pathogenesis (Grifoni et al., 2020; Ye et al., 2020). An imbalance in this ratio as is seen in serious cases of COVID-19 could be owed to T cell or B cell dysfunction, with the latter being reliant on T cell responses (Grifoni et al., 2020). Therefore, it is vital that the various roles of T cells in the context of SARS-CoV-2 infection are better understood, especially in regards to the aging immune system, the most apparent targets of severe disease.

At puberty, there is a 90% drop in T cell production via involution of the thymus, which turns to adipose tissue (Fulop et al., 2018; Nikolich-Zugich et al., 2020). T lymphocyte production is again decreased around 40–50 years of age where the remainder of the thymus further degenerates (Nikolich-Zugich et al., 2020). This secondary atrophy causes production of naïve T cells to drop to only 1% of its output before primary involution. Additionally, lymph nodes become less capable of storage, maintenance, and support of naïve T cells with advancing age (Nikolich-Zugich et al., 2020). To illustrate, TCR excision circles are used as markers of naïve T cell output and decreased exponentially by 95% in subjects 60 years of age compared to 25 years (Ponnappan and Ponnappan, 2011). However, total numbers of T cells show little decline with age except in the very elderly, and this is due to a compensatory increased peripheral T cell proliferation, called homeostatic proliferation accompanied by acquisition of activated/memory cell phenotypes (Ponnappan and Ponnappan, 2011; Sprent and Surh, 2011). This means that while the elderly may have similar lymphocyte levels to younger adults, their ability to mount an adaptive response to a novel antigen is significantly reduced from replacement of naïve T lymphocytes with antigen-independent experienced cells (Ponnappan and Ponnappan, 2011). This has serious implications in the context of a novel pandemic, as the elderly are less likely to be able to mount a rapid, high-affinity T cell effector and regulatory response.

#### Th1/Th2

The levels and proportion of differentiated CD4 T cells into Th1 and Th2 types and their associated cytokines are vital for elimination of a pathogen without incurring damage to the host tissue (Ponnappan and Ponnappan, 2011; Biasi et al., 2020; Ye et al., 2020). Th1 cells are notably proinflammatory and secrete cytokines such as IFN-γ, IL-1β, IL-2, and most pathologically in COVID-19, IL-6 (Biasi et al., 2020; Ye et al., 2020). Th2 cells have primarily anti-inflammatory function via IL-4 and IL-10 and attempt to attenuate Th1 pathways to avoid uncontrolled attack on the host (Biasi et al., 2020; Ye et al., 2020). The optimal ratio of Th1/Th2 differs by the pathogen, and failure of the host to adequately regulate these pathways may result in prolonged disease states or mortality (Ye et al., 2020). While the original SARS-CoV produced and was adequately eliminated by a predominately Th1 response, host success over the novel coronavirus seems to prefer attenuation of these proinflammatory processes (Ye et al., 2020). While patients of COVID-19 have elevated Th1 and Th2 cytokines, patients who ultimately survived had significantly higher levels of anti-inflammatory Th2 cytokines than proinflammatory Th1 cytokines (Biasi et al., 2020). To complement, patients who died of COVID-19 had features of the infamous “cytokine storm” with highly upregulated levels of a plethora of mostly proinflammatory Th1 cytokines (Ye et al., 2020).

The literature on levels and ratio of Th1 and Th2 CD4+ cells and associated cytokine levels in healthy older populations is conflicting. However, most sources report that Th2 responses are upregulated in healthy elderly populations and also predominate in early disease states, which is associated with elderly susceptibility to tuberculosis, influenza, and other infections (Ponnappan and Ponnappan, 2011; Har-Noy and Or, 2020). What is troubling with SARS-CoV-2 is that like many viruses, it has evolved mechanisms for evading the host immune system, replicating insidiously (Har-Noy and Or, 2020; Nikolich-Zugich et al., 2020). This initial Th2 predominance in elderly populations initially aids coronavirus replication until host mechanisms are rapidly overwhelmed by the pathogen and high viral titers (Har-Noy and Or, 2020). In response, patients that progress to critical illness, in particular the elderly, are believed to experience a rapid switch to Th1-predominating pathways, overloading the host with inflammatory cytokines that inadvertently harm host tissue (Ye et al., 2020). This initial Th2 preference followed by a rapid upregulation of Th1 pathways is the molecular basis of inflammaging and the increase in secretion of proinflammatory cytokines in an activated, aging immune system (Ye et al., 2020). In fact, most critically ill patients and those who succumbed, did not initially report severe symptoms in early stages of the disease; the conditions of these patients deteriorated suddenly with the appearance of a rapid and extreme Th1 response (Ye et al., 2020).

This situation is incredibly interesting, as children, women, and especially pregnant women are known to have higher levels of Th2 cytokines in relation to COVID-19 (Molloy and Bearer, 2020). However, it is believed that these groups are better able to coordinate the transition and attenuation of Th1 responses once the infection is detected, preventing unnecessary host damage (Molloy and Bearer, 2020). Unfortunately, elderly populations appear to lose the ability to smoothly transition and coordinate this ratio once viruses have been detected, which may explain the rapid cytokine storm and deterioration of hospitalized patients (Ponnappan and Ponnappan, 2011).

#### Th17

Another component of the adaptive immune system of interest in immune senescence is the Th17 cell type. These cells mainly produce proinflammatory cytokines such as IL-17 and IL-22 and are involved in the stimulation of IL-6 and TNF (Schmitt et al., 2013; Nikolich-Zugich et al., 2020). In cases of MERS, SARS, and COVID-19, secretion of Th17’s hallmark cytokine IL-17 is directly related to disease severity (Nikolich-Zugich et al., 2020). This is likely because in many coronavirus infections, IL-17 functions to recruit neutrophils; it additionally activates IL-22 and associated cytokines that cause pathological states and pulmonary edema in the host (Grifoni et al., 2020; Nikolich-Zugich et al., 2020). In SARS, IL-17 has also been found to be involved in proinflammatory activation of the complement cascade which activates mast cell degranulation in the lungs and is predictive for ARDS development (Nikolich-Zugich et al., 2020). Additionally, IL-17 is positively regulated by IL-6 which is significantly increased in serious states of COVID-19, adding to the fatal cytokine storm (Ponnappan and Ponnappan, 2011; Biasi et al., 2020; Ye et al., 2020). In a Chinese population, an IL-17 polymorphism analysis showed that patients predisposed to lower levels of IL-17 secretion had greater 30-day survival for ARDS compared to patients with genetically higher levels of IL-17 (Xie et al., 2019).

Studies have shown that Th17 cells and IL-17 are significantly higher in human and murine elderly populations (Ponnappan and Ponnappan, 2011; Schmitt et al., 2013; Lim et al., 2014). In a mouse model, Th17-associated proinflammatory cytokines IL-17, IL-1β, CCR6, CCL20, ROR-γt, and IL-22 were significantly upregulated even in healthy aged mice compared to young subjects (Lim et al., 2014). This increase in Th17 and IL-17 levels is especially present in elderly patients with autoimmune and/or inflammatory diseases, especially obesity (Liu and Nikolajczyk, 2019). Evidence shows that adipocytes, reservoirs for Th1, IL-6, and TNF, can directly activate differentiation of CD4 T cells into Th17 via the STAT3 pathway (Liu and Nikolajczyk, 2019). These Th17 cells are involved with a feed forward loop of adipose tissue inflammation which over the years can be a primary driver of systemic inflammation, heart disease, diabetes, fatty liver, and cancer (Liu and Nikolajczyk, 2019; Maffetone and Laursen, 2020). These conditions, especially type II diabetes, in turn provide unique positive feedback and support for Th17 production (Ip et al., 2016). This presents an interesting overlap, as a growing proportion of adults over 65 are overweight or obese, and up to 94% of patients with serious cases of COVID-19 had at least one underlying disorder where adiposity could be a trigger (Maffetone and Laursen, 2020). The immune responses of obese COVID-19 patients are largely unexplored and present a rich area for research development.

#### Treg Cells

Immunosuppressive regulatory T (Treg) lymphocytes function to inhibit inflammatory T cells including Th1, Th2, and Th17 cells. To protect host tissue, they secrete immune-suppressive cytokines such as IL-10 and TGF-β which antagonize the effects of the proinflammatory IL-6 and IL-17 that are implicated in COVID-19 (Ponnappan and Ponnappan, 2011; Schmitt et al., 2013; Ye et al., 2020). The optimal presence of Treg lymphocytes varies by disease but has been necessary for mild aftermath of MHV infections, while Treg diminution is associated with severe morbidity. Treg levels have not been sufficiently studied in the context of COVID-19, but a recent study reports an inverse correlation between naïve Treg levels and COVID-19 disease severity (Chen G. et al., 2020). Whether this relation has a causative role in disease outcome is yet unknown.

The literature on Treg function in the elderly is complicated. It seems that overall numbers of Treg cells in those over 65 years are significantly reduced compared to a 50 to 65-year age group (Schmitt et al., 2013). This difference is especially significant for elderly patients suffering from episodes of ischemic stroke (Dolati et al., 2018). This overall decline in Treg numbers is in line with the second wave of thymic degeneration, which may explain these numbers (Ponnappan and Ponnappan, 2011; Schmitt et al., 2013; Nikolich-Zugich et al., 2020). Foxp3 is a key transcription factor and marker of Treg cells, necessary for Treg function and inhibition of all CD4+ subtypes (Ponnappan and Ponnappan, 2011; Schmitt et al., 2013). While overall Treg numbers are lower, it seems that expression of Foxp3 is upregulated, giving the remaining Treg cells such strong CD4+ immunosuppressive potential that even anti-inflammatory cytokines such as IL-10 are inhibited (Ponnappan and Ponnappan, 2011; Schmitt et al., 2013). This increase in Treg immunosuppression is quite counterintuitive when realizing its effects on CD4 pathways that already function to attenuate inflammation (Schmitt et al., 2013). Additionally, although there is higher functioning of Treg cells, it seems that inhibition of the Th17 subtype is lost in elderly patients, producing a high, proinflammatory ratio of Th17/Treg cells (Schmitt et al., 2013).

In parallel to the relationship between Th17-associated inflammation and adiposity, there is also a rich association between Treg populations and fat accumulation (Liu and Nikolajczyk, 2019). With the progression of obesity, the proportion of Tregs in adipose tissue wanes (Liu and Nikolajczyk, 2019). The expression profiles and phenotypes of these lymphocytes also differ across fat levels in the host (Liu and Nikolajczyk, 2019). Visceral adipose tissue-resident Tregs of lean individuals produce high levels of anti-inflammatory IL-10 (Liu and Nikolajczyk, 2019), which upregulates Th2 pathways, antagonizing effects of IL-6 and TNF, a mechanism also supposed to occur in COVID-19 patients (Ye et al., 2020). However, this anti-inflammatory cytokine production decreases with increasing levels of adiposity (Liu and Nikolajczyk, 2019). What is also interesting is that high fat levels in the diet may alter expression of Treg cells towards proinflammatory states (Carlsen et al., 2009). To elucidate, mice models have shown that a high fat diet, especially in males, increases expression of NF-κb, a protein critically involved in DNA production and proinflammatory cytokine release in the aging immune system (Carlsen et al., 2009). NF-κb levels significantly increase in the elderly, and increased expression of this transcription factor occurs through several pathways (Ponnappan and Ponnappan, 2011; Polesso et al., 2017). Non-canonical upregulation has been shown to reduce suppressive function in Tregs and actively gear them towards proinflammatory states (Polesso et al., 2017). From this association, elderly patients with high adiposity seem particularly susceptible to low levels of functional, immunosuppressive Tregs (Carlsen et al., 2009; Polesso et al., 2017; Liu and Nikolajczyk, 2019). Their Tregs are likely prone to inflammatory cytokine release, although more studies need to be done to establish a causal relationship.

### CD8 T Cells

Cytotoxic CD8 T cells are lymphocytes that recognize peptides presented in the context of MHC class I to identify infected host cells, and therefore are a vital component of the adaptive immune system (Ponnappan and Ponnappan, 2011; Grifoni et al., 2020). In fact, a majority of recovered COVID-19 patients had developed some type of CD8 proinflammatory cytokine response to the coronavirus infection (Grifoni et al., 2020). Based on several hospital studies, it is believed the SARS-CoV-2 replicates within host cells due to functional exhaustion of these cytotoxic cells (Diao et al., 2020; Zheng et al., 2020). Clinically, this often presents as lymphopenia and expression marker studies have shown that the remaining cytotoxic lymphocytes manifest markers of exhaustion (Diao et al., 2020; Zheng et al., 2020). For example, PD1 and Tim3 are fatigue markers of CD8 T cells that were upregulated in ICU patients compared to mild cases (Diao et al., 2020). These markers are also highly expressed in patients progressing from prodromal to active signs of COVID-19 disease (Diao et al., 2020). Yet another study supported these findings of cellular exhaustion by study of the NKG2A receptor on CD8 cells and NK cells (Zheng et al., 2020). Expression of this receptor was upregulated in both cells and correlated with significantly lower percentages of IFN-γ+ CD8, IL-2+CD8 T cells, and granzyme B+ CD8 T cells which are secretion-specific subtypes (Zheng et al., 2020). In addition, studies report upregulated levels of cytotoxic granules such as granzyme B and perforin markers (Grifoni et al., 2020). This data altogether implies that the novel coronavirus stimulates extreme activation of CD8 T cells and their cytokines with resultant fatigue.

As related to cell exhaustion, there is evidence that the PD-1 and Tim-3 exhaustion molecules are upregulated in CD8 lymphocytes from healthy aged mice compared to younger controls (Lee et al., 2016). A result of this exhaustion was the inability to proliferate under antigen stimulation of the TCR, indicating the lower functioning of aged cytotoxic cells (Lee et al., 2016). CD8 T cell numbers as they pertain to age have been largely unexplored in humans in the context of COVID-19. However, results from a study of the original SARS-CoV in a monkey model may lend interesting insights. It was found that peripheral lymphocytes, specifically CD8 T cells, were overall significantly lower in elderly monkeys both before and after SARS-CoV infection (Clay et al., 2014). Younger monkeys had higher CD8 counts for the entirety of the study, indicating that they were better able to sustain these levels (Clay et al., 2014). Elderly monkeys had a drop in CD8 counts after five days post-infection, resulting in higher viral titers (Clay et al., 2014). Flow cytometry analysis additionally confirmed that not only were the cell numbers lower, but their function and responsiveness were also reduced (Clay et al., 2014).

### B cells, Plasma Cells, and Antibody Response

Differences in function of B cells and plasma cells have yet to be studied in the context of COVID-19 or coronavirus infections, but there are relevant changes in this part of the adaptive immunity that are relevant for any pathogen. From production of antibodies to the secretion of cytokines, B lymphocytes are vital mediators of the adaptive immune system (Ponnappan and Ponnappan, 2011; Nikolich-Zugich et al., 2020; Ye et al., 2020). Of these two functions, degeneration in the quality of secreted antibodies has been better studied in the context of aging (Ponnappan and Ponnappan, 2011; Nikolich-Zugich et al., 2020; Ye et al., 2020). Because of age-related bone marrow degeneration, there is a decrease in production of naïve B lymphocytes (Nikolich-Zugich et al., 2020). Although this does not necessarily affect levels of peripheral plasma cells, these lymphocytes are antigen-experienced and have limited capability of binding to a new antigen. Therefore, antibodies against new epitopes may take longer to produce and maybe of lower affinity (Ponnappan and Ponnappan, 2011; Snyder and Farber, 2019). This decline in affinity and therefore responsiveness has been studied in both murine and human models (Ponnappan and Ponnappan, 2011). Studies have revealed that decreased affinity is not just due to lowered production (Henry et al., 2019; Casadevall and Pirofski, 2020). This difference has been shown to be linked to lower rates of isotype switching and diminished somatic hypermutation in the elderly immune system (Henry et al., 2019). In relation to influenza, elderly individuals also have lower rates of spontaneous mutations in the variable region of plasmablast genes (Casadevall and Pirofski, 2020). Additionally, in a murine model, the loss of germinal centers in lymph nodes also has a contributory role in this affinity diminution (Casadevall and Pirofski, 2020).

### Memory T and B Cells

After a pathogenic encounter, a proportion of antigen-specific effector T and B cells persist and continue as memory cells, undergoing rapid proliferation upon subsequent encounters of the same or similar antigens (Har-Noy and Or, 2020; Nikolich-Zugich et al., 2020). These cells are the basis for vaccine efficacy, and a lack of strong affinity between effector cells and pathogen epitopes can result in poor long-term immunity (Nikolich-Zugich et al., 2020). Of particular interest are antigen-experienced tissue-resident memory cells (Trm) that are potent mediators of local immune response (Snyder and Farber, 2019; Nikolich-Zugich et al., 2020). Studies in mice and humans have shown that these Trm are phenotypically distinct and exhibit distinct transcriptional profiles compared to circulating memory cells (Szabo et al., 2019). CD4 and CD8 Trm express the CD69 marker of activation and CD8 Trm express the αE integrin CD103 marker of tissue retention (Snyder and Farber, 2019; Szabo et al., 2019). With increasing age, these memory cells persist and accumulate in the epithelium of almost all organs, including the lungs (Snyder and Farber, 2019). With aging, the Trm cells acquire aberrant proinflammatory phenotypes in response to pathogenic invasion, and murine studies have shown that the age-related increases in Trm numbers likely predispose older mice to poorer outcomes for communicable and non-communicable diseases (Ritzel et al., 2016).

Aging also influences peripheral circulating memory T and B cell populations. Regardless of pathogen, in aging humans and mice, there is reduced production of naïve T and B cells from the thymus and bone marrow, respectively, with relative increase in proportions of memory cells (Goronzy and Weyand, 2017; Yanes et al., 2017; Fulop et al., 2018). As a compensatory mechanism, to maintain immune homeostasis and lymphocyte populations relatively constant, naïve lymphocytes undergo lymphopenia-induced proliferation or homeostatic proliferation and acquire memory cell phenotypes and properties. In addition, memory cells also undergo intermittent homeostatic proliferation driven by homeostatic cytokines (Surh and Sprent, 2008; Sprent and Surh, 2011; Yanes et al., 2017). However, there are differences in these phenomena between mice and humans and between the CD4 and CD8 compartments. While these homeostatic mechanisms of proliferation contribute to the maintenance of the peripheral lymphocyte pool in humans (Stulnig et al., 1995; Huppert et al., 1998; Ponnappan and Ponnappan, 2011; Goronzy and Weyand, 2017), this is in contrast to T cell homeostasis in murine models, where memory cell proliferation cannot prevent the contraction of the CD4+ and CD8+ compartments (Yanes et al., 2017). Several studies have shown that circulating memory CD4+ T cell responses are maintained in older mice and humans (Stulnig et al., 1995; Stephan et al., 1996; Huppert et al., 1998; Ponnappan and Ponnappan, 2011; Goronzy and Weyand, 2017). CD8+ T cells also undergo homeostatic proliferation in humans via expansion of memory phenotype cells and memory stem cells (Goronzy and Weyand, 2017; Yanes et al., 2017). However, homeostatic proliferation of CD8+ T cells is not as efficient as that of CD4+ cells, causing the lymphocyte compartment to diminish with age (Goronzy and Weyand, 2017; Yanes et al., 2017). Additionally, there is evidence that the memory CD8+ T cells are much less diverse than the CD4+ counterparts (Goronzy and Weyand, 2017; Yanes et al., 2017). This is relevant because although lymphocyte numbers may appear to be relatively constant, memory cell proliferation can negatively impact the clonal size, TCR diversity, repertoire stability, and effector function (Goronzy and Weyand, 2017; Yanes et al., 2017). One study found that recurrent antigen presentation produced a restricted diversity of circulating memory T cells in people between 65 and 75 years of age, while people over 75 years were found to have lowered numbers of memory T cells with limited proliferative capacity (Har-Noy and Or, 2020). This lowered production past 75 years may be due to cellular senescence secondary to excessive replicative pressure (Goronzy and Weyand, 2017). These T cells are also at risk of becoming exhausted from this chronic stimulation, with loss of cytokine production, cytotoxicity, and other effector functions (Goronzy and Weyand, 2017). However, these secondary effects of memory T cell proliferation have still been largely unexplored and represent a unique area for further research.

Analogous to the age-related dysfunction in T cells, circulating B cells in mice and humans show substantial differences in numbers and distribution of cell types through age (Ponnappan and Ponnappan, 2011; Riley et al., 2017; Yanes et al., 2017; Ma et al., 2019; Frasca et al., 2020). In aging mice, the B lymphocyte compartment has reduced frequencies of naïve cells along with an increase of antigen-experienced cells, including the presence of a of phenotypically unique subset called age-associated B cells (ABCs) in old mice (∼2 years of age) (Riley et al., 2017; Yanes et al., 2017; Ma et al., 2019). These proinflammatory antigen-experienced cells accumulate with age and may inhibit growth and survival of pro-B lymphocytes as well as common lymphoid progenitors (CLP; Riley et al., 2017; Ma et al., 2019). While the full actions and repercussions of these cells are yet unknown, the differentiation of these cells is promoted by TLR7 signaling (Riley et al., 2017; Ma et al., 2019). This is relevant in the context of COVID-19, as TLR7 is implicated in the recognition and signaling events following SARS-CoV-2 infection (Moreno-Eutimio et al., 2020). In humans, antigen-experienced, class switched B cells that lack expression of IgD and the memory marker CD27, also known as double negative B cells represent the equivalent of mouse ABCs and have been identified in healthy aging, autoimmune diseases, chronic viral and parasitic infections and recently also in COVID-19 patients (Ma et al., 2019; Cancro, 2020; Woodruff et al., 2020). In addition to this cell type, homeostatic proliferation of memory B cells is also known to occur in humans (Ponnappan and Ponnappan, 2011; Yanes et al., 2017). However, it appears that there is strong individual-specific variation in the number and distribution of circulating total and memory B lymphocytes in humans (Breitbart et al., 2002; Crooke et al., 2019). Nevertheless, this proliferation of antigen-experienced B cells allows B lymphocyte numbers to remain relatively constant through the years (Ponnappan and Ponnappan, 2011; Riley et al., 2017; Frasca et al., 2020). In humans as well as murine models, serum immunoglobulin levels are either sustained or increased with age (Ponnappan and Ponnappan, 2011; Frasca et al., 2020). However, older individuals exhibit a decline in effective cytokine production and display aberrant antibody responses such as decreased affinity maturation, poor neutralization of antigens, as well as heightened production of antibodies prone to autoreactivity (Ponnappan and Ponnappan, 2011; Yanes et al., 2017; Ma et al., 2019; Frasca et al., 2020). Furthermore, normally expected to undergo apoptosis, these B cells of elderly adults escape apoptosis, leading to the accumulation of abnormal antigen- and cytokine-producing cells (Ponnappan and Ponnappan, 2011). Overall, although circulating memory cell numbers and antibodies are expected to increase proportionately with aging, they may contribute to dysfunctional responses to new infections. As the SARS-CoV-2 is a novel strain, and the virus-specific persistence, tissue specificity, and inflammation across age are still unknown, memory cells present a potent, unexplored area for COVID-19 pathogenesis.

## Tissue-Specific Immunity

### Thymus

The thymus is a primary lymphoid organ, the site of T cell development and the source of mature, naive T cells. Starting at puberty, the thymus begins to atrophy in a process called thymic involution (Fulop et al., 2018). As a result of this phenomenon, an individual’s ability to respond to novel pathogens decreases. This process of thymic involution may seem counter-intuitive, but there are a few possible reasons for why this happens. First, the thymus is very metabolically active because it is producing large numbers of cells, and the body tends to down-regulate metabolic activities as one ages in order to conserve energy (Fulop et al., 2018). This phenomenon also occurs in muscle tissue and bone marrow with aging. Second, by this age, an individual has likely encountered a majority of pathogens in their immediate environment. Thus, it is physiologically beneficial to halt energy expenditure on the production of new T cells and rely instead on memory cells that recognize previously encountered microbes (Fulop et al., 2018).

Thymic involution is a possible explanation for why the elderly tend to experience worse outcomes when infected with novel pathogens. CD8 T cells and CD4 Th1 cells are particularly important for fighting viruses. In the case of SARS-CoV-2, the adaptive immune response becomes even more important because this virus employs many mechanisms to evade the innate immune response (de Wilde et al., 2017) such as shielding PAMPs and blocking IFN signaling. Without the production of new T cell receptors (TCR), the individual is unable to mount an efficient attack against any new pathogens they may encounter.

### Bone Marrow

Bone marrow is the site of production of all hematopoietic cells and is also the location of the maturation of B cells. Bone marrow naturally contains adipocytes that are useful during adolescent growth and also serve a protective function (Ma and Fang, 2013). As a person ages, these adipocytes grow to almost entirely replace the bone marrow by the age of 30 years (Veldhuis-Vlug and Rosen, 2018). Some studies have found an association between marrow adipose tissue and impaired hematopoiesis – when bone marrow cells were irradiated in tissue with a larger amount of marrow adipose tissue, there was impaired reconstitution of blood cells (Ambrosi et al., 2017). Given this negative correlation between marrow adipose tissue and blood cell development, it is possible that this has an impact on the diminished immune response seen in the elderly.

### Lymph Nodes

Lymph nodes are important hubs for antigen recognition, T and B cell activation and proliferation. In both aging mice and humans, lymph nodes show dysregulated interactions between B and T cells, decreased numbers of T cells, aberrant lymphocyte movement, and a decrease in both the number and size of germinal centers (Turner and Mabbott, 2017b). These changes, in addition to the loss of definition of follicular regions and germinal centers, have a negative impact on the mounting of an adaptive response. The loss of follicular structure likely results from a few different age-related changes. First, there is a decrease in the follicular dendritic cell network (FDCs). These cells are very important for the maintenance of the germinal center reaction. FDCs retain immune complexes for presentation to B cells in order to promote proliferation in the germinal center and isotype switching to mount an appropriate response. Additionally, aging lymph nodes show less CXCL13 expression in the follicle. This chemokine, along with its receptor CXCR5, are important for the homing of naive B cells into the follicle of a lymph node, where it can detect its antigen and undergo activation with T cells before undergoing clonal expansion in the germinal center. In addition to these changes, a study found that aging lymph nodes show decreased numbers of fibroblastic reticular cells and lymphoid endothelial cells (Thompson et al., 2019). The fibroblastic reticular cells, in particular, are extremely important for maintaining naïve T cells and overall lymph node organization. This study found that aging lymph nodes in mice and rhesus macaques showed increased fibrosis via a thickening of the outer capsule, infiltration of the stroma with collagen, and a reduction in the total size of the lymph node. Overall, these microscopic changes pose a potential mechanism behind the inability of the aging lymph node to retain naive T cells that have recently migrated into it (Thompson et al., 2019). For novel T-dependent antigens like SARS-CoV-2, naïve T cell retention and movement into the follicle is necessary for the activation of B cells and for the correct response for viral eradication.

### Spleen

The spleen has an important role in combating pathogens in the blood and activating an appropriate immune response to these pathogens. Normally, marginal zone B cells in the spleen can move between the marginal zone and the follicle in order to detect antigens and then undergo a germinal center reaction to T-independent antigens. In aging mice spleens, B cells show decreased mobility between these two locations due to an increased response to the chemoattractant sphingosine-1-phosphate (S1P), which keeps them in the marginal zone (Turner and Mabbott, 2017a). Given that SARS-CoV-2 is a T-dependent antigen, this may not be relevant to the elderly response to SARS-CoV-2, but it is still an important consideration.

### Peripheral Tissues

One study identified macroscopic and microscopic changes in the lungs associated with COVID-19. Based on autopsy findings, some macroscopic changes included pleurisy, lung consolidation, pulmonary edema, and occasional increased lung weight (Hanley et al., 2020). Interestingly, two asymptomatic patients underwent surgical resection for adenocarcinoma and SARS-CoV-2 was found during the procedure. Associated microscopic changes included edema, pneumocyte hyperplasia, inflammation, and multinucleated giant cell formation. Another study conducted an autopsy of a COVID-19 patient (Xu Z. et al., 2020) and found diffuse damage to the alveoli and cellular fibromyxoid exudates in both lungs. These exudates, consisting of mucous, fibrous tissue, and immune cells, are masses that have left the blood vessel and are a sign of inflammation. Additionally, both lungs showed significant lymphocyte infiltration into the interstitium of the lungs and cytopathic changes associated with a viral infection – atypical enlarged pneumocytes with large nuclei, amphophilic granular cytoplasm, and prominent nucleoli in the intra-alveolar spaces (Xu Z. et al., 2020). In the right lung, hyaline membrane formation and desquamation of pneumocytes were found; these two features together indicate ARDS. Both of these features make it significantly harder for a patient to breathe. Type 1 pneumocytes rely on their squamous shape for rapid diffusion of oxygen into the capillaries and carbon dioxide out of capillaries. The formation of hyaline membranes increases the diffusion distance and makes rapid diffusion even more difficult. In the left lung of this patient, pre-ARDS symptoms were noted: pulmonary edema and hyaline membrane formation (Xu Z. et al., 2020).

An interesting feature that has been noted in many COVID-19 patients is lymphopenia, or low lymphocyte counts. A study of 99 COVID-19 patients in Wuhan showed a 35% decrease in total lymphocyte number (Astuti and Ysrafil, 2020). Another study (Schaller et al., 2020) that conducted multiple autopsies on COVID-19 patients found that many of them showed “patchy” lymphocyte infiltration into lung tissue, which suggests that the poor immune response may be due to a lymphocyte migration problem. An additional autopsy (Xu Z. et al., 2020) found low counts of CD4 and CD8 T cells in this individual with hyperactivation of Th17 cells and large amounts of cytotoxic granules in CD8 T cells. There is reason to believe that this hyperactivity and cytotoxicity may account for the extensive lung damage that many patients are experiencing.

Severe acute respiratory syndrome coronavirus 2 is unique in that it seems to have an extensive impact on not just the lungs, but also on the gastrointestinal tract and the blood vessels. One reason for the vascular inflammation could be that endothelial cells, those that line blood vessels, express the ACE-2 receptor. One study (Albini et al., 2020) found that SARS-CoV-2 can bind to this receptor and infect the endothelial cells, which leads to endothelial cell inflammation and damage and can ultimately result in vasculitis, disseminated intravascular coagulation, thromboembolism, and worse patient outcomes. Many of these patients also show an increase in D-dimer and fibrin breakdown products. D-dimer is a protein that is made when a blood clot has been degraded and fibrin is a molecule involved in blood clotting. The presence of these two together may suggest the increase in formation and degradation of blood clots in COVID-19 patients, which may be related to thromboembolism. In a similar fashion, enterocytes in the ileum and colon express ACE-2 (Gu et al., 2020). When SARS-CoV-2 enters through this receptor, it can infect the enterocytes and cause diarrhea and the associated pain and cramping that some COVID-19 patients experience. The exact mechanism for the infection is still unknown.

### COVID-19 and Co-morbidities, Aging, and Disease Severity

It is well documented that patients with a history of diabetes, hypertension, chronic lung disease, cardiovascular disease, and obesity experience more severe symptoms and worse outcomes in COVID-19 (Bassendine et al., 2020). Many of these conditions are commonly seen in elderly patients. Heart disease is the leading cause of death in the United States for those who are 65+ years old, followed closely by chronic low-respiratory disease in third and diabetes mellitus in sixth place (Heron, 2019).

One suggestion for the cause of these worse outcomes is related to the adipokine enzyme DPP4 (Bassendine et al., 2020). This is the enzyme that MERS-CoV uses to enter cells, but new models of the SARS-CoV-2 spike protein show it can bind to human DPP4 as well via the S1 domain of the protein. Additionally, DPP4 dysregulation has been seen in COVID-19 patients. The DPP4 enzyme is found in salivary glands, kidneys, liver, colon epithelial cells, capillaries, type 2 alveolar cells, and T, B, and NK immune cells. DPP4 enzymatic expression is higher in elderly people and in many individuals who have diabetes or metabolic syndrome. Additionally, in mouse models with diet-induced obesity, adipose tissue is a source of DPP4 (Bassendine et al., 2020). This upregulation could be a potential predictor of the severity of the disease as DPP4 may be the link between co-morbidities and poor clinical outcomes. Additionally, the wide expression of DPP4 could explain the variety of symptoms of COVID-19. Taken together, this evidence presents a potential mechanism for the relationship between pre-existing conditions, comorbidities, and worse outcomes.

Lymphoid tissues including the thymus, bone marrow, lymph nodes, and spleen show dysregulation with age. This has a negative impact on an individual’s ability to respond to novel antigens. Many COVID-19 patients are showing a combination of lymphopenia and hyperactive immune cells, which causes extensive lung damage. Additionally, gastrointestinal epithelial cells and vascular endothelial cells express the ACE-2 receptor, allowing for SARS-CoV-2 entry and resulting destruction. DPP4, the enzyme associated with MERS-CoV infection, may be a link between comorbidities and poor patient outcomes.

## Therapeutics and Vaccines

Increased mortality from COVID-19 has been observed in elderly populations, likely due to comorbidities, immunosenescence, and the associated proinflammatory state which occurs during the aging process (Franceschi et al., 2000; Shaw et al., 2013; Weyand and Goronzy, 2016; Fuentes et al., 2017; Fulop et al., 2018). While a robust immune response is needed to overcome COVID-19 infection, hyperactivation of the immune system may cause excessive inflammation and tissue damage (Du and Yuan, 2020). Therapeutic options that may benefit older adults include senolytic or immunosuppressant drugs, which aim to reduce the cytokine storm caused by overproduction of proinflammatory cytokines (Sargiacomo et al., 2020). Senolytics, also called “anti-aging drugs,” promote removal of senescent immune cells that accumulate over an individual’s lifetime and overproduce cytokines (Sargiacomo et al., 2020). While clinical trials are ongoing to evaluate their efficacy and safety, drugs such as rapamycin and azithromycin that possess senolytic effects have been investigated as possible treatments for COVID-19, and may especially benefit older adults (Ma and Fang, 2013; Rea et al., 2018; Omarjee et al., 2020; Sargiacomo et al., 2020). Although clinical trials are still ongoing and more evidence is needed to confirm the risks and benefits of such drugs, immunoregulation through cytokine inhibition has been identified as possible treatment for COVID-19, and may provide significant advantages to older patients experiencing higher levels of cytokine-mediated inflammation (Ma and Fang, 2013; Rea et al., 2018; Omarjee et al., 2020; Sargiacomo et al., 2020). Rapamycin in particular was shown to improve viral clearance of H1N1 in animal models through down-regulation of senescent T-cells and reduction of cytokine storm, suggesting that its application in COVID-19 may similarly improve viral clearance, although more clinical data is needed to explore its use (Omarjee et al., 2020). Other drugs such as JAK-STAT pathway inhibitors and corticosteroids that inhibit cytokine signaling or otherwise dampen inflammation may also counteract hyperinflammation, especially in older patients with COVID-19 (Rea et al., 2018; Gozzetti et al., 2020; Jamilloux et al., 2020; Sargiacomo et al., 2020).

### IL-6 Inhibitors

Interleukin-6 is a pro-inflammatory cytokine that functions in neutrophil and cytotoxic T cell trafficking. Although the recruitment of immune cells may aid in virus control, the release of ROS and leukotrienes by neutrophils during the immune response may damage lung parenchyma and paradoxically lead to poor clinical outcomes. Evidence suggests that IL-6 dysregulation, as in the case of severe or chronic viral infections, may negatively impact lung function and contribute to hypoxemia (Vardhana and Wolchok, 2020). Elevated serum IL-6 has been observed in COVID-19 patients, with higher levels associated with more severe disease presentation and mortality. As a result, IL-6 dysregulation may negatively impact recovery. High serum IL-6 has also been found in older adults as part of the “inflamm-aging” phenotype, predisposing elders to more severe COVID-19 presentation (Rea et al., 2018).

As of November 2020, there exists no consensus across international guidelines on the treatment of COVID-19 patients with anti-IL-6 monoclonal antibodies (mAbs). The use of anti-IL-6 receptor mAb tocilizumab in COVID-19 treatment is supported by recommendations by both the Chinese Health Commission and the Italian Society for Infectious and Tropical Diseases. However, the Infectious Diseases Society of America (IDSA) recommends against tocilizumab treatment of hospitalized patients due to the lack of conclusive evidence demonstrated by clinical trials (Jamilloux et al., 2020).

Clinical trials assessing the use of anti-IL-6 mAbs in COVID-19 patients indicate some improvement to clinical outcomes, but are limited in sample size and experimental design heterogeneity. A case report of COVID-19 patients found that all three study participants showed resolution of fever, improvement of respiratory symptoms, and decreased C-reactive protein (inflammatory marker) post-treatment (Di Giambenedetto et al., 2020). A second study by Xu X. et al. (2020) found that among 21 severe or critical patients treated with tocilizumab, 75% showed decreased ventilation needs, 90.5% had improvement in lung pathology as observed via CT scan, and 100% showed clinical improvement. Although the study design lacked a control group, the results were compared to a previous study indicating a baseline mortality rate of 60% in critical patients and 11% in severe patients, suggesting potential benefits to tocilizumab administration. A third study yielded similar findings in 30 severe and rapidly declining COVID-19 patients (Roumier et al., 2020). A control group receiving standard of care was matched to the treatment group by age, gender, and disease severity. The tocilizumab group showed reduced need for mechanical ventilation and displayed a trend, albeit no statistically significant difference, towards lower mortality. One instance of ventilator-acquired pneumonia was observed, as well as 2 reports of mild hepatic cytolysis. Despite promising results, the lack of consistent treatment administration presented as a source of bias. Two patients receiving tocilizumab were also treated with hydroxychloroquine (HCQ) and azithromycin, while another two patients received methylprednisolone along with tocilizumab. Thus, tocilizumab may improve disease severity in COVID-19 patients, although more research is needed to evaluate appropriate timing and dosage.

Siltuximab is another anti-IL-6 mAb that displays higher affinity for IL-6 than tocilizumab. However, siltuximab is less studied than tocilizumab, and unlike tocilizumab, is not FDA-approved for management of cytokine release syndrome (Jamilloux et al., 2020). A compassionate-use study of 21 patients with COVID-19-related ARDS found that seven days after siltuximab treatment, 33% of subjects showed improvement, 43% stabilized (no change), and 24% had worsened condition. One death and one cerebrovascular event were observed (Jamilloux et al., 2020). While siltuximab has been identified as an alternate method of treatment for COVID-19, the lack of control group limits the ability to draw conclusions about its efficacy.

Aside from mAbs, antibiotics such as azithromycin serve as another method of IL-6 inhibition. Azithromycin promotes degradation of senescent cells, in addition to inhibiting IL-1 and IL-6 (Sargiacomo et al., 2020). This effect can be observed in patients with cystic fibrosis, in which azithromycin degrades myofibroblasts and decreases lung inflammation. Azithromycin may work synergistically with HCQ to inhibit viral replication in COVID-19 (Andreani et al., 2020; Ulrich and Pillat, 2020). However, current evidence suggests more harm to benefit regarding azithromycin and HCQ combination treatment (Boulware et al., 2020; Kamp et al., 2020; Recovery Collaborative Group et al., 2020).

### Chloroquine/Hydroxychloroquine

Severe acute respiratory syndrome coronavirus 2 enters cells via one of two receptors: CD147 and ACE2 receptor. The antimalarial drug chloroquine (CQ) and its less toxic analog, HCQ, have been shown to modify the glycosylation of the ACE2 receptor, thus limiting viral entry into the host cell (Liu et al., 2020). Moreover, HCQ has been shown to have anti-inflammatory effects, suggesting that it may help diminish the production of pro-inflammatory cytokines. Preliminary evidence suggested that HCQ and azithromycin, an antibiotic belonging to the macrolide class, may work synergistically to decrease viral load both in cell cultures and *in vivo* (Andreani et al., 2020). These drugs are not without risk, however. HCQ, CQ, and azithromycin may prolong the QT interval and induce cardiac arrhythmias (Kamp et al., 2020). As HCQ and CQ both possess a narrow therapeutic index, overdose and cardiotoxic effects are likely. There have been several reports of fatality in COVID-19 patients due to adverse cardiac events (Kamp et al., 2020). Despite much sensationalism in the media purporting the therapeutic effects of HCQ, clinical trials have yielded poor results that suggest more harm than benefit. In randomized controlled trials, HCQ has not been found to work effectively as prophylaxis or treatment for COVID-19 (Boulware et al., 2020; Recovery Collaborative Group et al., 2020). At this time, the use of hydroxychloroquine is not recommended for COVID-19 treatment due to lack of substantial evidence regarding its benefits. We anticipate that the results of an ongoing Phase IIb clinical trial by the US National Institutes of Health and another trial by the WHO may clarify the efficacy of CQ and HCQ in treatment of COVID-19 patients.

### mTOR-NLRP3-IL-1β Inhibitors

The NOD-like protein receptor 3 (NLRP3) inflammasome is another potential target for cytokine regulation. NLRP3 produces inflammatory cytokines IL-1β and IL-6 in response to cellular stresses such as ROS, which are released by mitochondria as signs of oxidative damage (Omarjee et al., 2020). Inflammasome activation is increased during the aging process due to compromised autophagy and impaired “cellular housekeeping” which facilitates ROS accumulation. Activation of the NLRP3 inflammasome is regulated by mTOR, which is in turn inhibited by rapamycin. The upregulation of NLRP3 and mTOR during SARS-CoV-2 infection leads to lung inflammation and fibrosis via IL-1β production.

Rapamycin, also known as sirolimus, reduces IL-1 and IL-6 production. An analog of rapamycin decreased serum IL-6 and improved T cell function in older adults with T cell senescence, indicating that rapamycin may be suitable for use in older COVID-19 patients (Omarjee et al., 2020). Moreover, early rapamycin treatment was associated with greater H1N1 influenza clearance, lower duration of ventilation, and lower rates of hypoxemia and multiple organ failure. Early rapamycin may yield similar positive effects in COVID-19 infection. Currently, two clinical trials assessing use in COVID-19 patients are underway – Efficacy and Safety of Sirolimus in COVID-19 Infection, and Sirolimus Treatment in Hospitalized Patients with COVID-19 Pneumonia or SCOPE, which may further inform the use of rapamycin in COVID-19 patients.

### Corticosteroids

Corticosteroids are anti-inflammatory agents that dampen the adaptive immune response. This may be beneficial because a stronger adaptive immune response is associated with longer time to improvement and higher rates of complications such as end-organ damage (Du and Yuan, 2020). The presence of a pro-inflammatory state in older adults suggests a need to modulate balance between viral clearance and excessive inflammation, which may be achieved using corticosteroids (Rea et al., 2018). A mathematical model of SARS-CoV-2 infection proposes that the adaptive immune response onset, which happens after that of innate immunity, occurs before the virus infects the maximum number of target cells (Du and Yuan, 2020). As a result, a stronger response may prolong the disease progression and increase opportunity for secondary consequences, while a weaker response may shorten the disease course due to rapid viral spread and depletion of available host cells. Mathematical modeling predicts that temporary, early administration of corticosteroids to delay the adaptive immune response will shorten the time to viral load peak from 2–3 week to 8–14 days post-exposure (Du and Yuan, 2020). A shorter disease course may reduce risk of end-organ damage and viral spread to other body parts such as the heart and kidneys. A strong adaptive immune response may additionally cause a “second wave” in which patients improve for a short time but quickly decline once the immune system is exhausted.

Glucocorticoids were commonly used to reduce systemic inflammation in SARS patients during the 2003 epidemic, suggesting that glucocorticoids may be a means of reducing similar symptoms in COVID-19 patients (Zhang W. et al., 2020). However, risks concerning use of corticosteroids to treat COVID-19 include impaired viral clearance and higher rates of secondary bacterial or fungal infections. Studies on corticosteroid use in COVID-19 patients yield conflicting results. A prominent clinical trial from the RECOVERY Collaborative Group found that administration of dexamethasone reduced mortality in COVID-19 patients receiving mechanical ventilation (The WHO Rapid Evidence Appraisal for COVID-19 Therapies (React) Working Group et al., 2020). Retrospective studies of hospitalized COVID-19 patients similarly suggest that early, low-dose corticosteroid use in patients with SARS-CoV-2 and SARS-CoV may lower mortality (Zhao et al., 2003; Wang et al., 2020). However, a meta-analysis of corticosteroid use in SARS patients with variable timing and dosage of treatment found overwhelming harmful effects, including increased length of stay in an intensive care unit, higher rate of secondary infection, and higher mortality (Russell et al., 2020). The COVID-19 Global Rheumatology Alliance has also identified increased COVID-19 hospitalization rates among people taking glucocorticoids, further indicating possible risks with corticosteroid use (Gianfrancesco et al., 2020). Moreover, studies examining treatments for coronaviruses such as MERS-CoV have suggested minimal to no benefit to steroid therapy. A retrospective observational study of 309 adults with critical MERS found that corticosteroid treatment did not result in a statistically significant difference in 90-day mortality, but was associated with delayed clearance of viral RNA (Arabi et al., 2017). However, difficulties in assessing the validity of these studies include the possibility of immortal time bias and indication bias from time-varying confounders. Immortal time bias refers to the condition that patients survive long enough to complete treatment, leading to positive bias, while indication bias from time-varying confounders refers to the increased likelihood of alternative therapies to be administered to critically ill patients after first-line therapies have failed, leading to improper assumption of negative treatment effects. Due to conflicting evidence and the presence of potential bias, additional information is required to elucidate the role of corticosteroid use in COVID-19 patients.

### JAK-STAT Pathway Inhibitors

The Janus kinase (JAK) inhibitors ruxolitinib and baricitinib have been identified as possible therapeutic agents against COVID-19. Although JAK inhibitors may reduce cytokine storm and improve COVID-19-induced ARDS, current literature is limited by the small sample size of studies and poor experimental design. For example, ruxolitinib is commonly used to suppress cytokine production in inflammatory diseases such as myelofibrosis and graft versus host disease (La Rosée et al., 2020). Although not without risk of adverse events due to opportunistic infections, its pro-apoptotic and anti-inflammatory effects on senescent cells may benefit elderly patients, who undergo immunosenescence over the course of the aging process (Gaspari et al., 2020). Studies have suggested that ruxolitinib may improve respiratory function and reduce need for mechanical ventilation in patients with ARDS (Gaspari et al., 2020; Gozzetti et al., 2020; La Rosée et al., 2020). However, these studies only analyze data from a small number of patients. Therefore, additional trials are needed to confirm these results.

Baricitinib is another JAK inhibitor that has been identified as a potential therapeutic option for COVID-19. As with ruxolitinib, preliminary studies have shown some benefit to baricitinib therapy, but this finding awaits validation by additional evidence and larger sample sizes (Cantini et al., 2020; Caputo et al., 2020). Baricitinib inhibits viral entry into cells by inhibiting AP2-associated protein kinase-1 (AAK1), which regulates receptor-mediated endocytosis of SARS-CoV-2 (Cantini et al., 2020). Both type I (IFN-α and IFN-β) and type II (IFN-γ) IFNs participate in the JAK-STAT pathway and serve as potential therapeutic targets. Type 1 IFN plays a key role in viral infections because it limits viral replication, promotes virus clearance, and stimulates tissue repair (Jamilloux et al., 2020). The importance of type I IFN in the antiviral response has led to concerns about the use of JAK inhibitors in COVID-19 treatment. In older adults, immunosenescence and the associated reduction in type I IFN and may contribute to greater disease severity (Spinelli et al., 2020).

Type I IFN has been studied in the response to the closely related virus MERS-CoV (Jamilloux et al., 2020). While IFN 1βb therapy has yielded some success in animal models, it failed to improve the course of disease in humans (Omrani et al., 2014; Arabi et al., 2020; Jamilloux et al., 2020). Analysis of type 1 IFN in COVID-19 suggests that SARS-CoV-2 degrades type I IFN mRNA and thus inhibits the type 1 IFN response (Ribero et al., 2020). High IFN-α and IFN-β signaling was observed in patients with mild to moderate COVID-19 while low IFN-α and IFN-β was observed in patients with severe COVID-19 symptoms, suggesting that overcoming type I IFN blockade may lead to milder symptoms (Acharya et al., 2020). Delayed type I IFN response is associated with decreased virus clearance and may increase overall hyperinflammation as a compensatory measure (Jamilloux et al., 2020). Impaired IFN facilitates leukocyte accumulation and apoptosis of T cells and endothelial cells, which in turn may contribute to ARDS by causing damage to alveoli and microvasculature (Jamilloux et al., 2020). However, IFN-α/IFN-β therapy has been shown to only improve outcomes when administered in the early inflammatory phase, as it yields negative effects when administered in the late phase (Seif et al., 2020).

Limitations in existing type I IFN clinical studies include small sample size, differences between studies in experimental designs and clinical conditions, and difficulties in assessing whether outcome was due to type I IFN alone or the drugs used in combination (Jamilloux et al., 2020). Only one retrospective study investigating the use of type I IFN therapy alone exists so far (Zhou Q. et al., 2020). However, significant flaws include the absence of a control group, inclusion of subjects with only moderate COVID-19 symptoms, inclusion of predominantly young and healthy subjects, and heterogeneity in treatment administration.

### Remdesivir

Several therapeutic candidates have been identified which aim to suppress the cytokine storm associated with COVID-19. Older adults, who experience systemic chronic inflammation due to the natural aging process, may benefit from such therapeutic options. However, conflicting evidence has prevented a consensus from being reached about the efficacy of these options.

Remdesivir, a nucleoside analog that inhibits viral replication in SARS-CoV and MERS-CoV, has been identified as a promising candidate in COVID-19 treatment. Preliminary results from a double-blind, randomized, controlled trial suggest that remdesivir may effectively decrease time to recovery (Beigel et al., 2020). The median recovery time in the remdesivir group was 10 days, compared to 15 days in the placebo group. Remdesivir was associated with lower rates of severe adverse events, including respiratory failure. While the mortality rate showed a trend towards lower fatality in the remdesivir group (7.1% compared to 11.9%), this difference was not statistically significant. Based on these findings, preliminary evidence supports the treatment of COVID-19 patients with remdesivir. However, the success of remdesivir has been contradicted by emerging evidence from the WHO Solidarity trial, a randomized controlled trial that has garnered much attention for its large enrollment number of over 11,000 participants. A preprint version of the Solidarity trial results found little difference between the in-hospital mortality, ventilation need, and length of hospital stay of patients treated with remdesivir and standard-of-care (Consortium et al., 2020). More evidence is needed to reconcile the conflicting findings of these studies. Therapeutic approaches to specifically address the needs of the elderly population may use additional agents in combination with remdesivir - although this approach has yet to be assessed.

### Vaccines

According to the WHO, 47 vaccine candidates have entered the clinical evaluation stage of development while 155 vaccine candidates have entered pre-clinical evaluation.^3^ The vaccines under development include live attenuated vaccines, inactivated virus vaccines, sub-unit, viral vector-based, DNA, and RNA vaccines. Each vaccine technology offers different advantages and disadvantages. Of the ten vaccines listed by the WHO as undergoing phase III trials, three candidates are formulated as inactivated virus vaccines, four as viral vector vaccines, two as mRNA vaccines, and one as a protein subunit vaccine. We anticipate that the FDA may soon approve vaccines for use in the US, as many Phase III clinical trials are underway or are nearing completion.^4^ Both Moderna Inc. and Pfizer Inc./BioNTech SE have reported promising results from the Phase III studies of their respective mRNA-based vaccine candidates. A press release published on November 18, 2020, reports that the primary efficacy analysis for the vaccine put forth by Pfizer/BioNTech has demonstrated 95% efficacy against COVID-19 among all study participants and 94% efficacy in adults over 65 years of age.^5^ The vaccine developed by Moderna has similarly shown 94.5% efficacy in the interim analysis of Phase III trials.^6^ Both vaccines have been shown to be well-tolerated with few reported adverse events.

Inactivated virus vaccines, which contain killed virions, have been previously tested and their efficacy and safety has been shown in phase I studies against the SARS-CoV coronaviruses (Lin et al., 2007; Kaur and Gupta, 2020). In addition, because the viral particles have lost their pathogenicity, inactivated virus vaccines do not confer the risk of causing disease in immunocompromised individuals, which may occur with live attenuated vaccines (Zhang et al., 2019). However, inactivated vaccines may produce lower titers of antigen-specific antibodies and require booster shots to maintain efficacy over time (Kaur and Gupta, 2020). The inactivated vaccine produced by Sinovac is currently in phase III trials and has shown seroconversion rates of up to 90% after 14 days post-immunization in phase I trials (Kaur and Gupta, 2020). Conversely, live attenuated vaccines contain live virions that have been weakened. Because live attenuated vaccines allow the immune system to encounter live pathogens, they typically induce high immunogenicity that does not require maintenance through booster shots unlike their inactivated counterparts (Kaur and Gupta, 2020). A limitation of live attenuated vaccines, however, is the need for additional measures during development due to the risk of mutations that revert to virulence and cause disease (Zhang et al., 2019).

As opposed to inactivated and live attenuated vaccines that contain whole pathogens, subunit vaccines include antigenic fragments and may include adjuvants to increase immunogenicity. However, subunit vaccines typically only confer short-term immunity with questionable induction of memory immunity, and generally produce less effective CD8+ responses (Zhang et al., 2019; Kaur and Gupta, 2020). The NVX-CoV2373 vaccine produced by Novavax is another candidate in phase III evaluation and is formulated with recombinant COVID-19 S-Protein.^7^ Trials in animal models have shown that a single dose has been sufficient to produce high immunogenicity with anti-spike protein antibodies (Kaur and Gupta, 2020).

Viral vector-based vaccines use vaccine vectors to express and deliver antigens to host cells. A benefit to this model is the decreased involvement of infectious particles, which may aid in the development process (Kaur and Gupta, 2020). However, disadvantages include the possibility that host immunity may neutralize the vector before it is able to deliver the antigen, thereby reducing efficacy of the vaccine (Kaur and Gupta, 2020). Carcinogenic effects may also occur due to possible integration of viral genome into the host genome (Kaur and Gupta, 2020). Ad5-nCoV vaccine developed by CanSino Biologics in partnership with the Beijing Institute of Biotechnology is a recombinant adenovirus type-5 vector expressing the SARS-CoV-2 spike protein.^8^ Phase I clinical trials indicated that the vaccine was effective in producing S protein-specific neutralizing antibodies (Kaur and Gupta, 2020). Phase III trials are underway.^9^

DNA vaccines allow transcription of antigen and adjuvant, which mimics the course of infection with live virus. Advantages to DNA vaccines are induction of both humoral and cell-mediated immune responses, as well as temperature stability (Kaur and Gupta, 2020). However, DNA vaccines have historically evoked poor cytotoxic and humoral immunity and may not be as effective as other formulations. Safety concerns include integration of viral DNA into host genome, which may activate oncogenes and increase cancer risk (Zhang et al., 2019).

RNA vaccines utilize similar delivery technology as DNA vaccines, but do not pose the risk of integration into the host genome (Zhang et al., 2019). Like DNA vaccines, they allow for expression of antigenic material, which models infection with a live virus. However, they may be comparably more unstable than DNA viruses and may cause reactogenicity, or inflammatory responses to vaccination (Kaur and Gupta, 2020). The vaccine mRNA-1273, developed by the pharmaceutical company Moderna, has garnered attention after being fast-tracked by the FDA through Phase II trials and demonstrating 94.5% efficacy in preliminary results from the Phase III study https://investors.modernatx.com/news-releases/news-release-details/modernas-covid-19-vaccine-candidate-meets-its-primary-efficacy. Pfizer/BioNTech has similarly developed an mRNA vaccine candidate, which has shown 95% efficacy among all enrolled Phase III study participants and 94% efficacy in adults over 65 years old.^10^

The high efficacy rate demonstrated by the Pfizer vaccine in older adults is especially promising, as vaccination in adults over 65 has typically proven less effective due in part to aging-related immunosenescence. Diminished production and differentiation of naive B and T cells may contribute to decreased responsiveness to immunization, and weaker efficacy of influenza, hepatitis, and pneumococcal vaccination has been observed in older adults (Seidman et al., 2012; Vermeiren et al., 2013; van Werkhoven et al., 2015; Nikolich-Zugich et al., 2020). In response to influenza vaccination, older adults develop lower numbers of CD4+ memory cells, CD8+ T cells, and express less CD80 when compared to younger adults (Poland et al., 2014). Older populations may also produce weaker antibody- and cell-mediated responses to vaccines, including fewer IgA and IgM antibodies and diminished lymphocyte proliferation due to telomere shortening over time.

Strategies to improve immunization efficacy include booster doses and vaccine adjuvants (Lord, 2013). An *in vivo* study of rhesus monkeys observed that a second vaccination one month after the first dose boosted antibody titers to levels comparable to those found in young adults, suggesting that booster shots may help compensate for decreased vaccine responses in the elderly. Adjuvanted vaccines, which include substances that produce heightened immune response, may additionally help to compensate for less aggressive antibody and T cell signaling due to aging. The addition of TLR5 ligand flagellin in influenza vaccines was shown to increase efficacy and improve the TLR5-mediated immune response. The addition of glycoprotein E to the herpes zoster subunit vaccine was also shown to produce more robust immunity in older patients (Lal et al., 2015). The adjuvanted subunit vaccine yielded a 96.6–97.9% efficacy across all age groups, while the live attenuated vaccine Zostavax produced the following efficacy rates: 51.3% in adults aged 60 years or older, 69.8% in adults between 50 and 59 years, and 37.6% in adults over 70 years (Lal et al., 2015). As a result, the adjuvanted vaccine produced a substantial increase in efficacy, with the greatest difference observed in adults over 70 years of age. No increase in serious adverse events was observed compared to placebo.

A study of incomplete Freund’s adjuvant (IFA) containing mineral oil and surfactant similarly favored the use of adjuvanted vaccine and indicated that an oil formulation may further enhance efficacy (Lian et al., 2020). The oil emulsion in IFA-containing immunizations better distributed both ovalbumin (antigen) and lipopolysaccharide (adjuvant), causing stronger Th1 differentiation in response to vaccination. As a result of increased Th1 levels, higher secretion of TNF-α and IFN-γ secretion was observed. Moreover, higher levels of OVA+ monocytes and CD8α+ DCs were found post-immunization when compared to the vaccination without IFA.

Older populations experience severe COVID-19 symptoms at disproportionate rates and stand to benefit the most from development of a suitable vaccination. While immunosenescence may cause decreased vaccine efficacy in the elderly, appropriate development of vaccines may help ameliorate this effect. Adjuvanted vaccines with oil instead of saline formulation may confer a more robust immune boost and greater protection against SARS-CoV-2.

Both Pfizer and Moderna have developed lipid nanoparticle-encapsulated modified mRNA vaccines shown to induce strong humoral immunity (Kaur and Gupta, 2020). The mRNA-1273 vaccine developed by Moderna encodes for the full-length, stabilized SARS-CoV-2 spike protein, while the BNT162b1 vaccine developed by Pfizer/BioNTech encodes for the receptor binding domain of the spike protein and the T4 fibritin-derived foldon trimerization domain of the RBD antigen (Kaur and Gupta, 2020). The preliminary results of the Phase III clinical studies of both mRNA vaccines represent exciting advancements in the sphere of vaccine development, as the mRNA vaccine is relatively novel technology enabling efficient delivery of the genetic code producing the SARS-CoV-2 antigen to the intracellular target site (Zhang et al., 2019). The use of lipid nanoparticles to deliver self-adjuvanted mRNA formulated with cell penetrating peptides such as protamine, or to deliver self-amplifying mRNA containing the RNA-dependent RNA polymerase complex genome, have been shown to generate high antibody titers without the addition of adjuvants (Zhang et al., 2019). As the BNT162b1 vaccine has demonstrated similar efficacy in study participants over 65 and overall, we anticipate that developments made in the near future may further our understanding of the efficacy of vaccine technologies in both the general and elderly populations.

## Conclusion

The COVID-19 pandemic has revealed a striking demographic bias in the number of cases and deaths, with the elderly population being the most significantly afflicted. Besides the impact of genetics and underlying comorbidities, aging causes numerous physiologic changes within the immune system broadly categorized into immunosenescence and inflamm-aging. These factors cause progressive decline in the immune system’s ability to fight latent and novel infections and mount adequate responses to vaccines, while promoting pro-inflammatory phenotypes and therefore affect not only an individual’s susceptibility to coronavirus infections but also determine the disease course and clinical outcomes. Better understanding of these factors is necessary to tailor therapies and vaccine strategies in a step towards personalized medicine especially for deadly pandemic-causing diseases like COVID-19.

## Author Contributions

VM conceptualized the review. VB, NG, AS, and VM prepared the figures. All authors performed literature reviews, synthesized the information and wrote the manuscript.

## Conflict of Interest

The authors declare that the research was conducted in the absence of any commercial or financial relationships that could be construed as a potential conflict of interest.

## Footnotes

1 www.who.int/news-room/commentaries/detail/covid-19-and-the-use-of-angiotensin-converting-enzyme-inhibitors-and-receptor-blockers

2 www.clinicaltrials.gov/ct2/show/NCT04364893

3 www.who.int/publications/m/item/draft-landscape-of-covid-19-candidate-vaccines

4 https://www.fda.gov/emergency-preparedness-and-response/coronavirus-disease-2019-covid-19/covid-19-vaccines

5 www.pfizer.com/news/press-release/press-release-detail/pfizer-and-biontech-conclude-phase-3-study-covid-19-vaccine

6 www.investors.modernatx.com/news-releases/news-release-details/modernas-covid-19-vaccine-candidate-meets-its-primary-efficacy

7 www.who.int/publications/m/item/draft-landscape-of-covid-19-candidate-vaccines

8 www.who.int/publications/m/item/draft-landscape-of-covid-19-candidate-vaccines

9 www.who.int/publications/m/item/draft-landscape-of-covid-19-candidate-vaccines

10 www.pfizer.com/news/press-release/press-release-detail/pfizer-and-biontech-conclude-phase-3-study-covid-19-vaccine
